# Identifying Methylation Pattern and Genes Associated with Breast Cancer Subtypes

**DOI:** 10.3390/ijms20174269

**Published:** 2019-08-31

**Authors:** Lei Chen, Tao Zeng, Xiaoyong Pan, Yu-Hang Zhang, Tao Huang, Yu-Dong Cai

**Affiliations:** 1 School of Life Sciences, Shanghai University, Shanghai 200444, China; 2 College of Information Engineering, Shanghai Maritime University, Shanghai 201306, China; 3 Shanghai Key Laboratory of PMMP, East China Normal University, Shanghai 200241, China; 4 Key Laboratory of Systems Biology, Institute of Biochemistry and Cell Biology, Chinese Academy of Sciences, Shanghai 200031, China; 5 Institute of Image Processing and Pattern Recognition, Shanghai Jiao Tong University, and Key Laboratory of System Control and Information Processing, Ministry of Education of China, Shanghai 200240, China; 6 IDLab, Department for Electronics and Information Systems, Ghent University, 9000 Ghent, Belgium; 7 Shanghai Institute of Nutrition and Health, Shanghai Institutes for Biological Sciences, Chinese Academy of Sciences, Shanghai 200031, China

**Keywords:** breast cancer, subtype, methylation, pattern, multi-class classification

## Abstract

Breast cancer is regarded worldwide as a severe human disease. Various genetic variations, including hereditary and somatic mutations, contribute to the initiation and progression of this disease. The diagnostic parameters of breast cancer are not limited to the conventional protein content and can include newly discovered genetic variants and even genetic modification patterns such as methylation and microRNA. In addition, breast cancer detection extends to detailed breast cancer stratifications to provide subtype-specific indications for further personalized treatment. One genome-wide expression–methylation quantitative trait loci analysis confirmed that different breast cancer subtypes have various methylation patterns. However, recognizing clinically applied (methylation) biomarkers is difficult due to the large number of differentially methylated genes. In this study, we attempted to re-screen a small group of functional biomarkers for the identification and distinction of different breast cancer subtypes with advanced machine learning methods. The findings may contribute to biomarker identification for different breast cancer subtypes and provide a new perspective for differential pathogenesis in breast cancer subtypes.

## 1. Introduction

According to epidemiological statistics provided by the World Health Organization in 2015, more than 8.8 million deaths, accounting for one in six deaths, are attributed to cancer [1]. Cancer has become one of the major threats to human health. Among different cancer sites from different tissues and organs of the human body, breast cancer is one of the most common in women, with more than 1.7 million new cases recorded in 2012, accounting for one in four new cancer cases among all female cancers worldwide [2,3]. This disease has also been identified as one of the top five common causes of deaths, generating more than 0.57 million deaths in 2015 [1]. Therefore, breast cancer is widely regarded as a severe disease for humans worldwide, especially for women.

Histologically, breast cancer refers to all cancers that develop from breast tissues. Major risks for such disease include active risks (risks that patients can actively avoid) and passive risks (risks that patients can only passively experience) [4]. The major active risks for breast cancer are dietary patterns, obesity, and lack of childbearing [5], and the major passive risks for breast cancer are biological sex (women with higher mobility), genetic background, and age [6,7]. Among these risk factors, genetic background has recently received attention due to the research progression in cancer biology and the development of next-generation sequencing [8]. Various genetic variations, either hereditary or somatic mutations, contribute to the initiation and progression of breast cancers [9]. Genes such as *TP53* [10], *HER2* [11], *BRCA1,* and *BRCA2* [12] are related collectively or independently to breast cancer pathogenesis. Genes such as *BRCA1* and *BRCA2* [12] are even named after particular subtypes of breast cancer, indicating their unequivocal genetic contribution. In general, the major clinical symptom for the initiation and progression of breast cancer is an abnormal region on the breast that feels differently from the rest of the breast tissues [13]. A physical breast exam is the first step in breast cancer diagnosis. The two common diagnostic approaches for further medical testing and verification of breast cancer are mammograms (e.g., low-dose X rays) and lump biopsies [13,14,15]. Identifying accurate and sensitive biomarkers during cancer detection through blood or biopsy samples is essential. According to histological and biochemical studies, non-specific breast cancer markers such as carcinoembryonic antigen (CA) 15-3, and CA 27.29 have been identified as potential biomarkers for breast cancer at the protein level [16,17]. However, these conventional biomarkers have limited clinical applications because they are also identified in other tumor subtypes and even in healthy people who are under stress. In addition, these biomarkers cannot be distinguished from breast cancer subtypes with different pathogenic mechanisms and corresponding treatments. With the development of liquid biopsy and high-throughput sequencing technologies, the detection of breast cancer biomarkers has been extended to the system level [9,17,18,19,20]. Hence, the diagnostic parameters of breast cancer are no longer limited to the protein content and can include newly discovered genetic patterns such as CNV [9], methylation [21], and microRNA [22]. Correspondingly, the task and ability of breast cancer detection extend to the detailed subtyping of breast cancer (e.g., disease or treatment stratifications) to provide subtype-specific indications for further personalized treatment [23,24].

In general cancer studies, cancer epigenetics refers to all the studies on multiple epigenetic modifications to the cancer cell DNA [25]. General cancer epigenetics studies focused on the pathogenic significance of somatic DNA methylation, histone modification and microRNA gene silencing processes. There are three major pathological mechanism for such modification to contribute to tumorigenesis: (1) abnormal gene expression regulation, (2) dysfunctional DNA repair pathways and (3) pathological chromosomal instability. The abnormal epigenetic modification has been identified to be more frequently than other kinds of pathological characteristics in tumors like somatic mutations [25,26]. Therefore, the screening for epigenetic markers of tumors may be one of the major part of basic and clinical study of tumor diagnosis and treatment [27]. Among all such patterns of cancer epigenetics modification, abnormal DNA methylation patterns turn out to be some of the most frequent and significant pathogenesis for various cancer subtypes. Cancer genomes have been shown to be hypo-methylated comparing to adjacent normal cells’ genome [26]. The hypo-methylation pattern of cancer genomes is generally triggered by dysfunctional DNA methyl-transferases and may further lead to promoted mitotic recombination and damaged chromosomal structures [26]. Such pathological epigenetic modification may contribute to the tumorigenesis and has been widely identified in multiple cancer subtypes. Apart from such general influences, the abnormal methylation of some specific region on the genome may also be quite important for the trigger of tumorigeiesis. For instance, genes like *BRCA1*, *CDH1*, *RARB2*, *PTEN* have all been reported to have abnormal methylation epigenetically modified DNA fragments in the promoter or exonic regions and such modifications have also been confirmed to participate in the tumorigenesis in breast cancer [28,29]. Therefore, considering that some abnormal methylation patterns/markers are not only specific enough for the identification of tumor cells but also be essential for tumorigenesis, it is quite necessary for the screening of specific epigenetic especially methylation markers in tumors as potential clinical markers that guiding the diagnosis and treatment of specific tumor subtypes.

To date, machine learning-based methods have been widely used for analyzing biological and biomedicine data [30,31]. Model et al. applied feature selection for high-dimensional methylation data to classify different cancers [32], showing selecting the right number of features using feature selection is crucial for cancer classification. Adorjan et al. applied supervised and unsupervised machine learning methods to disseminate tumors using the CpG sites [33]. Chen et al. applied feature selection and supervised classifier to classify samples from different MSI statuses in colorectal cancer using expression data [34]. Shipp et al. applied supervised machine learning models to classify diffuse large B-call lymphoma with expression profiles of 6817 genes [35]. Similarly, Ye et al. trained supervised classifiers to predict hepatitis B virus–positive metastatic hepatocellular carcinomas based on the expression profiles [36]. For methylation profiles, machine learning-based methods can also give useful hints. 

One genome-wide expression–methylation quantitative trait loci analysis confirmed that different breast cancer subtypes (i.e., basal, Her2, LumA, and LumB) have varying methylation patterns [21]. According to the statistics provided by the World Health Organization, for different races, the proportion of different subtypes in all breast cancer patients are different. In Asian or Pacific populations, more than 50% of breast cancer cases turn out to be LumA subtype and the basal-like subtype only accounts for 5%. However, in Hispanic populations, basal-like subtype accounts for 11.6% and in African-American populations, this subtype accounts for more than 30%. No matter in which population, LumA subtypes always represent the vast majority, always accounting for more than 40%. As for Her2 and LumB subtypes, in each population, such two subgroups always account for 30% and LumB subtypes accounts for twice comparing to Her2 subtypes [37]. Considering the complicated subgrouping pattern of breast cancer and the imbalanced distribution patterns, it is quite significant to extract key biomarkers for the detailed and accurate subgrouping of breast cancer. Although various potential differentially methylated genes have been identified, the number of potential clinical biomarkers is extremely high. As mentioned above, machine learning-based methods may give considerable help. In this study, we attempted to screen a small group of functional biomarkers for the identification and distinction of different breast cancer subtypes using some advanced machine learning methods. The findings may contribute to the biomarker identification for different breast cancer subtypes and provide a new perspective for the differential pathogenesis in breast cancer subtypes.

## 2. Results

In this study, we first ranked the input 436,506 methylation features by using the MR score and then selected features with relevance scores larger than 0.2. In total, 9777 highly relevant features were kept for subsequent experiments. The MR scores for individual features are provided in Supplementary Table S1. The obtained 9777 features were analyzed by the MCFS method, producing a feature list provided in Supplementary Table S2. 

**Figure 1:**
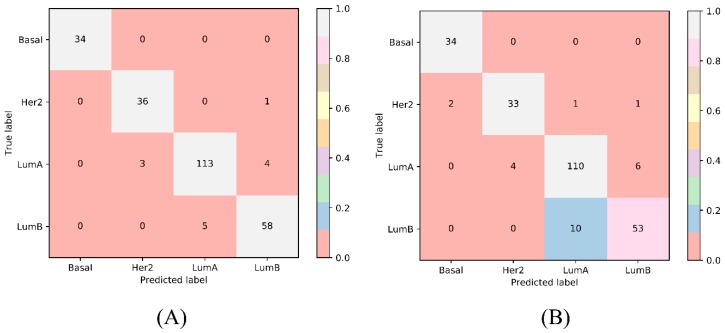
The confusion matrix yielded by the best support vector machine (SVM) and random forest (RF) classifiers. (**A**) The confusion matrix of the best SVM classifier; (**B**) The confusion matrix of the best RF classifier.

**Figure 2:**
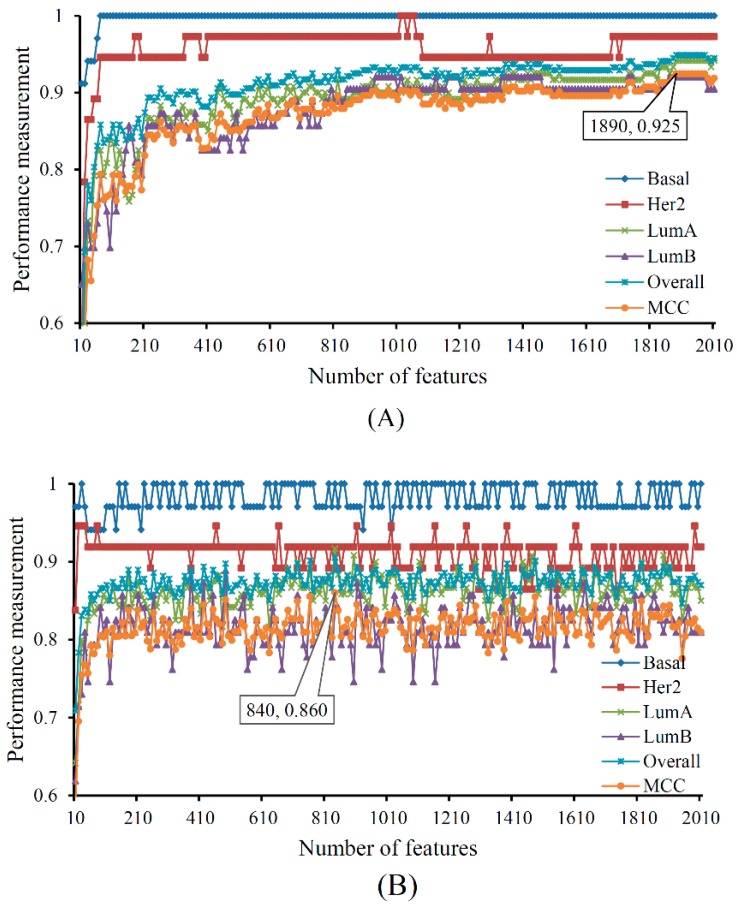
The performance of support vector machine (SVM) and random forest (RF) change with the number of features. (**A**) The performance of SVM; (**B**) The performance of RF.

To further select discriminative features with a supervised classifier, we ran incremental feature selection (IFS) with a multi-class support vector machine (SVM) to classify samples from four breast cancer subtypes. A series of feature subsets was generated with a step interval of 10, and the SVM was trained and evaluated on the training samples consisting of the features from each feature subset by using a 10-fold cross-validation. The result yielded the best Matthews correlation coefficient (MCC) of 0.925 and an overall accuracy of 0.949 when using the top 1890 features (Table 1). These features are termed as optimum features and constitute the optimum feature set. The sensitivity and specificity of each class yielded by the SVM with optimum features are also listed in Table 1. Each of them exceeds 0.9, indicating the good performance of such SVM classifier and the importance of the optimum features. The performance on minority classes (basal, Her2, LumB) is also high, suggesting the utility of Synthetic Minority Over-Sampling Technique (SMOTE). The corresponding confusion matrix yielded by such SVM classifier is illustrated in Figure 1A. Supplementary Table S3 details the information of the top 1890 features, and Supplementary Table S4 reports the performances of the SVMs corresponding to all feature subsets. With these performance measurements, an IFS curve was plotted (Figure 2A) with the performance, including sensitivity of each class, overall accuracy and MCC, as *y*-axis and the number of features as *x*-axis.

Other supervised multi-class classifiers exist, for example random forest (RF) [38,39,40]. To verify the power of SVMs, we ran IFS with integrated RF for each feature subset in the same way and trained and evaluated RF on samples consisting of features from individual feature subsets by using a 10-fold cross-validation. When the top 840 features were used, RF achieved the best MCC value of 0.860 and an overall accuracy of 0.906 (Table 1). The sensitivity and specificity of each class yielded by such RF classifier are also listed in Table 1. None of them exceed the corresponding measurements yielded by the best SVM classifier. The corresponding confusion matrix is shown in Figure 1B. 

Supplementary Table S5 provides the performances of RF for individual feature subsets, and Figure 2B illustrates that the performance of RFs changed with the number of used features. These results reveal that the SVM is a good choice for classifying breast cancer samples from basal, Her2, LumA, and LumB, thereby verifying it as the supervised classifier for IFS in this work.

## 3. Discussion

### 3.1. Analysis of Top Ranked Genes

We identified a group of functional genes with different methylation status in various breast cancer subtypes. According to recent publications, the top-10 ranked identified genes with distinctive methylation status have been confirmed, thereby validating the efficacy and accuracy of our prediction. The detailed analysis and discussion of each functional gene are presented below. 

In our prediction list, *NTHL1*, which encodes a functional DNA *N*-glycosylase of endonuclease III family, has been predicted to have differential methylation status in various breast cancer subtypes. Although no direct report has confirmed the detailed methylation status of such a gene in breast cancer, a study in 2014 reported that breast cancer with differential breast cancer mutational status can be clustered into various subtypes with different *NTHL1* expression patterns; such expression level distinction of *NTHL1* is probably induced by epigenetic regulation [41]. Therefore, we speculate that in different molecular subtypes of breast cancers, the differential methylation status of *NTHL1* should be associated with its differential expression and could represent a substantial epigenetic modification pattern [42], thereby validating the efficacy and accuracy of our prediction. According to functional annotation and enrichment, the identification of such gene indicated that glycolysis and gluconeogenesis may be alternative pathogenic biological processes for different breast cancer subtypes. 

The next predicted gene is *CMBL*, which encodes a specific cysteine hydrolase of the dienelactone hydrolase family and normally has high expression in liver cytosol but not in breast tissues [43,44]. In 2014, an early study on proteasome function in breast cancer confirmed that cysteine hydrolase has an abnormal expression level in certain breast cancer subtypes, and this finding corresponds with our prediction [45]. In 2016, another study confirmed that cysteine hydrolases may be functionally related to proteolytically activated receptors, and the genes encoding these hydrolases such as *CMBL* may have differential expression patterns and biological roles in particular breast cancers subtypes with distinctive epithelial–mesenchymal transition (EMT) tendency [46]. Therefore, *CMBL* may also be a potential biomarker for breast cancer subtyping. 

The gene *FLJ43663* is functionally related to an important breast cancer associated gene, *ESR1* [47], but its methylation status has not been directly confirmed in breast cancer subtypes. However, as the clone of *LINC-PINT*, the methylation of this gene has been functionally related to multiple tumor subtypes [48,49]. In 2017, a study on the breast cancer subtyping of Chinese Han women confirmed that the methylation status of the gene *FLJ43663* underlying the expression pattern of *LINC-PINT* is functionally related to the initiation and progression of Luminal A subtype of breast cancer but not of other subtypes [50], thereby validating the efficacy and accuracy of our prediction. 

The next predicted gene is *LPP*, which encodes a member of LIM domain protein subfamily contributing to the regulation of cell-cell adhesion and cell motility [51,52]. This gene has pathogenic contribution on breast cancer at methylation level. In 2018, one study on the epigenetic regulation of LIM family genes in different breast cancer subtypes confirmed that the putative promoter region of *LIMD1* and our predicted gene *LPP* may be abnormally methylated in the MDA-MB435 cell line [53]. Further studies on the expression pattern or methylation status of this gene confirmed its abnormal methylation pattern only in breast cancer with high metastatic tendency but not in all samples, thereby indicating that its methylation status may be an effective subtyping biomarker for particular breast cancer patients [54,55]. 

For the following predicted gene, *ANP32B* is a cell survival factor and participates in cell cycle progression [56,57]. According to a study on *ANP32B* in mouse model, a high expression of this gene may promote the progression of breast cancer [56]. Considering the specific regulatory role of methylation on gene expression pattern, the demethylation of *ANP32B* may contribute to tumorigenesis in breast cancer. Another study in 2016 reported that not all breast cancer subtypes can be induced by the abnormal methylation or expression of our predicted gene *ANP32B* [58], thereby indicating the conditional methylation status of this gene contributes to different breast cancer subtypes. 

The next predicted gene *ZCCHC24* encodes a functional zinc finger protein, which is functionally related to platelet biosynthesis and body height [59]. One study on *ZCCHC24* in 2017 confirmed that this gene may participate in tumorigenesis by inhibiting the biological function of BET family proteins [60]. Early in 2016, another study confirmed that different breast cancer subtypes may have various pharmacological reactions against BET inhibitors, thereby reflecting the distinctive contribution of BET signaling pathways in different breast cancer subtypes [61]. Therefore, the expression pattern of *ZCCHC24* in different breast cancer subtypes may be functionally different, and its specific methylation pattern affecting its expression level may also be different and discriminative. These findings validate the efficacy and accuracy of our prediction.

In addition to *ZCCHC24*, another zinc finger protein coding gene named *ZNF282* has also been predicted as a potential distinctive marker for different breast cancer subtypes. As a specific regulator and binder of U5 repressive element, this gene contributes to breast cancer as an estrogen receptor co-activator but not in other pathogenic mechanisms [62]. Hence, the methylation status and expression pattern of *ZNF282* may be different in breast cancers with high and low expression levels of estrogen receptor. The expression of estrogen receptor is one of the clinical diagnostic and subgrouping biomarkers for breast cancer [63,64,65]. Therefore, our predicted gene *ZNF282* can be regarded as an additional biomarker related to estrogen receptor for breast cancer subtyping. 

As the next predicted gene associated with breast cancer subtyping, *SFT2D2* contributes to the fusion of retrograde transport vesicles [66]. Although only a few descriptive studies have been published about this gene, breast cancer *SFT2D2* has been confirmed to contribute to metastatic pathogenic behaviors [67]. The methylation status and expression pattern of this gene vary in breast cancer subtypes such as basal and HER2-like ones, thereby validating its potential distinctive function in molecular subtyping of breast cancer.

*BCL9* is a specific gene functionally related to B-cell acute lymphoblastic leukemia and participates in Wnt/β-catenin and GPCR signaling pathways [68]. According to recent publications, *BCL9* is a specific breast cancer associated gene that contributes to the invasion and EMT of breast ductal carcinoma but not of other subtypes, thereby indicating its potential subtyping relevance [69]. Another study on *BCL9* in breast ductal carcinoma confirmed that its methylation status and expression pattern are definitely functionally related to the expression of *ERBB2* and *HER2* in breast cancers [70]. Considering that *ERBB2* and *HER2* are both confirmed molecular subtyping biomarkers for breast cancer [71], the functional links of the two genes with our predicted gene indicate that *BCL9* may also be a potential biomarker at the methylation level, thereby validating the efficacy and accuracy of our prediction. Classifying this gene as potential biomarker also confirms the specific pathogenic role of beta-catenin binding processes in different breast cancer subtypes. 

As a specific member of the sorting nexin family, *SNX1b* contributes to the regulation of cell-surface expression of epidermal growth factor receptor [72]. *SNX1b* and its homologues participate in breast cancer tumorigenesis by mediating *BRMS1*-dependent transcriptional repression [73]. Breast cancer subtypes with high metastatic tendency have a low methylation status and a high expression pattern of this gene [74], thereby indicating the potential sub-grouping relevance of *SNX1b* with its pathogenic contribution to different breast cancer subtypes.

In summary, all predicted optimal genes with high rank (the top 10) have been confirmed to participate in breast cancer associated biological processes and have various methylation status and biological roles in different breast cancer subtypes. On the one hand, these identified genes may be potential clinical biomarkers in biopsy samples for breast cancer subtyping. On the other hand, they contribute to different pathogenic mechanisms for various breast cancer subtypes, thus establishing a panorama for breast cancer tumorigenesis at the conditional cellular and molecular levels.

### 3.2. Case Study on LumB

The SVM with optimum features provided good performance as listed in Table 1, from which we can see that the sensitivity on LumB was lowest. It is interesting to investigate whether the SVM can provide better performance if LumB samples are removed. This section gave the results on such study and made further analysis. 

**Figure 3:**
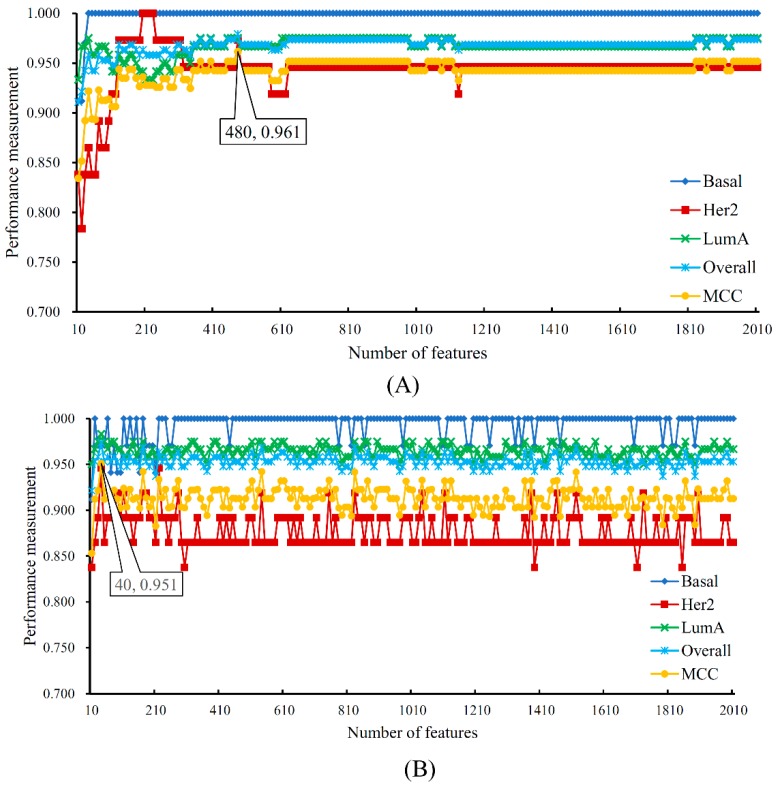
The performance of support vector machine (SVM) and random forest (RF) change with the number of features on the dataset without LumB samples. (**A**) The performance of SVM; (**B**) The performance of RF.

Samples in other three breast cancer subtypes: Basal, Her2 and LumA, were represented by 9777 features, which were analyzed by the MCFS method. All 9777 features were sorted in the decreasing order of their RI scores. Obtained feature list is provided in Supplementary Table S6. Similarly, an IFS with SVM was applied on this feature list. The performance of SVM on different feature sets is provided in Supplementary Table S7. The IFS curve was plotted in Figure 3(A). The highest MCC was 0.961 when top 480 features were used. The detailed performance of such SVM is listed in Table 2. 

Compared with the results on the whole dataset (Table 1), when LumB samples were excluded, the performance of SVM improved and it can be achieved using much fewer features. These cases also occurred for RF, please see Figure 3B, Table 2 and Supplementary Table S8. 

**Figure 4:**
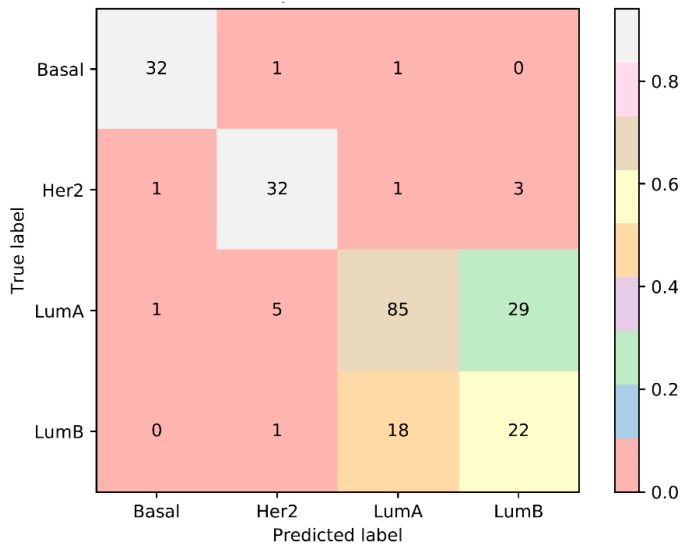
The confusion matrix yielded by the SVM with top 40 features.

As mentioned above, when LumB samples were excluded, the performance of SVM and RF improved and much fewer features were used. It is necessary to investigate the reason why this phenomenon occurred. By checking the results of IFS with SVM on the dataset without Lumb samples (Supplementary Table S7), the MCC arrived at 0.922 when top 40 features were adopted. It was a little lower than that obtained by the SVM with optimum features on the whole dataset, which was 0.925 (Table 1). On the othe hand, the MCC obtained by the SVM with top 40 features on the whole dataset was only 0.655, which was much lower than 0.922. The sensitivities on Basal and Her2 were all high (higher than 0.860), while those on LumA and LumB were much lower (about 0.700). In addition, by checking the confusion matrix, as shown in Figure 4, among 120 LumA samples, 35 samples were misclassified, where 29 samples were classified to LumB; for 63 LumB samples, 19 samples were not correctly classified and 18 samples were assigned to LumA. All these indicated that LumA and LumB samples were quite similar, inducing difficulities to identify LumB samples from LumA samples. LumA and LumB are two subtypes of luminal breast cancer. Luminal breast cancer can be identified by three major parameters named as estrogen receptor (ER), progesterone receptor (PR) and human epidermal growth factor receptor (HER-2). The typical molecular characteristics of luminal breast cancer turn out to be either ER or PR positive together with HER negative. Among the top 40 features (probes), thirty-one probes can be annotated onto the potential functional genes, several of which have been validated to have different methylation patterns or expression levels that possibly controlled by epigenetic factors by recent publications. These features can effectively distinguish luminal breast cancer from other breast cancer subtypes, but cannot perfectly distinguish different luminal breast cancer subtypes. We picked up some features to confirm such a fact. 

A probe named as cg24921140 detected the 3’UTR region of *SSR3*, implying the specific expression alteration of such gene in different subtypes of breast cancer. *SSR3* encodes a glycosylated endoplasmic reticulum membrane receptor and has been reported to contribute to the regulation of calcium metabolism [75,76]. The expression of *SSR3* has been confirmed to be correlated with the biological effects of *HER2*, *Ki-67* and *TP53* [77,78]. As we all know, luminal subtype of breast cancer refers to a subtype of breast cancer with either ER (estrogen receptor) or PR (progesterone receptor) positive together with HER-2 (human epidermal growth factor receptor) negative. Considering the differential expression pattern of *HER2* and *Ki-67* mentioned above, it is reasonable to imply that the expression level of *SSR3* may also contribute to the distinction of luminal breast cancer from other breast cancer subtypes.

*ALDH3B1*, as another target for the identified probe cg18174530, has been generally reported as a major regulator in the detoxification of aldehydes [79,80]. As for its specific methylation pattern in different breast cancer subtypes, according to recent publications, such a gene has been confirmed to be differentially methylated and expressed in luminal breast cancer subtypes [81]. The next probe, cg13345122, targets another effective gene, named as *STK39*. According to recent publications, this gene has been confirmed to function in the cellular stress response associated processes [82,83]. As for the methylation pattern of such gene in different breast cancer subtypes, it has been confirmed that the expression level of such gene varies in basal and luminal breast cancer samples, indicating the potential distinctive effects of such gene for breast cancer subtyping [84]. Considering that epigenetic silencing like methylation has been reported to be one of the most effective processes for the regulation of *STK39* expression, it is reasonable to speculate that such probe targeting *STK39* may also distinguish luminal subtypes from other breast cancer subtypes [85]. However, no direct reports confirmed the differential methylation pattern of such gene in different subtypes of breast cancer, revealing the inapplicability of such probe on distinguishing LumA and LumB subtypes.

As for the probe targeting effective gene called cg04326566, it targets a member of the homeodomain family of NDA binding protein coding gene, named as *CUX1*. According to recent publications, such gene contributes to the regulation of neuronal differentiation in the brain [86,87]. As the upstream of FGF1/HGF signaling pathway, *CUX1* has been reported to contribute to the progression of luminal and basal breast cancer, help distinguishing Her2 subtype of breast cancer [88]. Apart from that, further study on cell-based assays confirmed that such gene has different expression pattern in basal breast cancer subtype comparing to the luminal subtypes [89]. 

As for the probe cg12642725, targeting the 5’UTR of *RERG*, was also identified. *RERG* encodes a member of the RAS superfamily participating in the regulation of cell proliferation and tumor formation [90,91,92]. As for its specific role in different breast cancer subtypes, early in 2011, *RERG* has been screened out as a specific biomarker for ER-positive luminal, like breast cancer subtype [91].

As discussed above, several top features can be validated to distinguish luminal breast cancer from other breast cancer subtypes. However, they cannot further distinguish different luminal breast cancer subtypes (LumA and LumB), inducing difficulties for SVM to identify LumA and LumB samples. In addition, we further investigated the confusion matrices for top 10–100 features, which are available in Supplementary Table S9. The same phenomenon also occurred. Thus, the SVM with small numbers of features cannot provide good performance on LumB because LumA and LumB samples were too similar to identify them.

**Figure 5:**
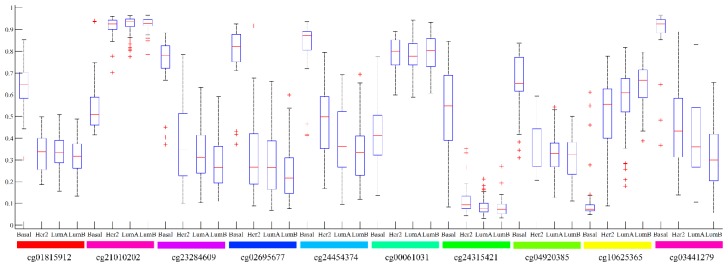
Boxplots to illustrate the distributions of top ten features on four breast cancer subtypes.

Besides, for top ten features on the whole dataset, we investigated their distributions on four breast cancer subtypes, which are shown in Figure 5. It can be observed that for amlost all features, their distributions on LumA were most similar to those on LumB, which further confirmed the poor performance of SVM with top features on LumB, as discussed above. The distributions on Her2 followed and those on basal were most different. 

## 4. Materials and Methods

### 4.1. Datasets

We downloaded the methylation profiles of 34 basal, 37 Her2, 120 LumA, and 63 LumB breast cancer patients from the Gene Expression Omnibus under the accession number of GSE84207 [21]. The methylation profiles of 436,506 probes were measured with Illumina Human Methylation 450 Bead Chip, and the methylation levels were represented with beta values. We investigated the methylation patterns of different breast cancer subtypes and whether or not these subtypes can be rediscovered by using methylation profiles. 

### 4.2. Feature Selection

**Figure 6:**
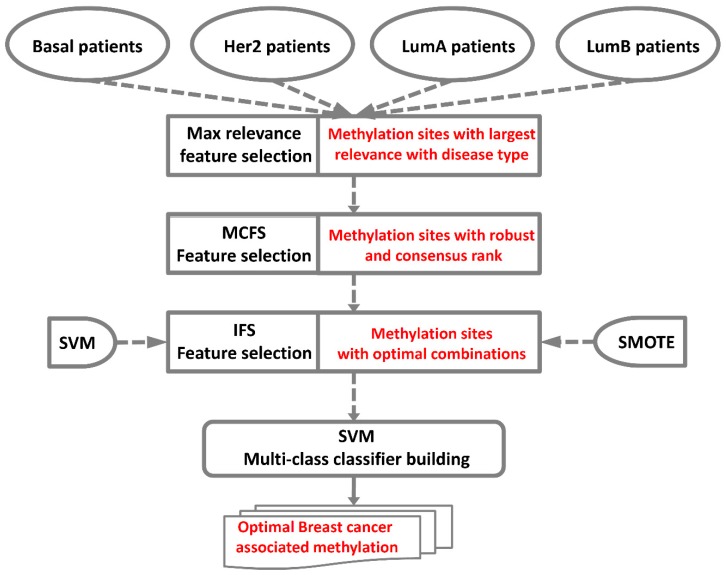
Flowchart for classifying samples from four breast cancer subtypes.

Figure 6 shows a two-step feature selection strategy, including combining maximum relevance (MR) feature selection [93,94,95,96,97,98,99,100,101,102] and Monte Carlo feature selection (MCFS) [9,103,104,105,106] to rank input features (e.g., methylation sites). The top-ranked features were further fed into the IFS with SVM to classify samples from four breast cancer subtypes, for example basal, Her2, LumA, and LumB.

#### 4.2.1. Maximum Relevance Score

To select the relevant features to output labels (e.g., breast cancer types), we calculated relevance scores, which are a part of the maximum relevance and minimum redundancy feature selection method [93,94,95,96,97,98,99,100,101]. The relevance score is defined as the mutual information between label *x* and feature *y*:

(1) $$I(x,y)=\iint p(x,y)\log\frac{p(x,y)}{p(x)p(y)}dxdy$$

where *p*(*x*) and *p*(*y*) are the marginal probability density for *x* and *y*, respectively, and *p*(*x*, *y*) is the joint probability density. When this value is high, the relevance of this feature to class label is also high, implying that this feature is important for classification. By setting a threshold for the relevance score, several important features can be obtained. 

#### 4.2.2. Monte Carlo Feature Selection

MCFS is a supervised feature selection method based on multiple decision trees and bootstrap sets [9,103,104,105,106,107]. First, *p* bootstrap sets, which are randomly sampled from the original training set with replacement, are generated, and *t* feature subsets (each subset includes *m* features from the original *M* features and *m* is much smaller than *M*) are then produced. One decision tree is grown from one combination of the bootstrap sets and feature subsets; hence, *p* × *t* decision trees should be grown in total. 

An important feature should be selected as node feature with high choice for growing decision trees on the basis of which relative importance (RI) score can be calculated for individual features. This score is calculated depending on several factors, including the number of splits where this feature is involved in all nodes of the total *p* × *t* trees, the weight for each split according to its information gain during tree construction, the number of samples corresponding to this split node, and the classification accuracy of the final whole decision tree. The formulation of RI score for feature *g* is:

(2) $$RI_{g}={\sum_{\tau =1}^{pt}\left(wAcc\right)}^{u}\sum_{n_{g}(\tau )}IG(n_{g}(\tau )){\left(\frac{\mathrm{no}.\;\mathrm{in}\;n_{g}(\tau )}{\mathrm{no}.\;\mathrm{in}\;\tau }\right)}^{v}$$

where $n_{g}(\tau )$ represents the nodes in tree τ on which the split is made on feature *g*, $IG(n_{g}(\tau ))$ denotes the gain information of $n_{g}(\tau )$, $(\mathrm{no}.\;\mathrm{in}\;n_{g}(\tau ))$ denotes the number of samples in node $n_{g}(\tau )$, (no. in τ) represents the number of samples in the root of tree τ, wAcc stands for the weighted accuracy of tree τ, and u and v are the two regular factors. Clearly, a feature with a high RI score is important for classification.

In this study, we used MCFS program downloaded from http://www.ipipan.eu/staff/m.draminski/mcfs.html. For convenience, this program was executed with its default parameters. *u* and *v* are set to one. After obtaining the RI score for each feature, we sort features in the decreasing order of their RI scores. The obtained feature list is formulated by:

(3) $$F=[f_{1},f_{2},\ldots ,f_{n}]$$

where *f_i_* denotes a feature and *n* represents the number of investigated features.

#### 4.2.3. Incremental Feature Selection

Optimum features are required to obtain the best classification performance for distinguishing four breast cancer subtypes, which were further selected by an IFS with an integrated SVM classifier [94,96,103,104,108,109,110,111]. With the feature list *F*, a series of feature subsets is generated with a step interval of 10, in which the first feature subset has the top 10 features, and the second feature subset has the top 20 features and so on. We denote these feature subsets as $F_{10},F_{20},F_{30},\ldots$, where the subscript of *F* indicates the number of features included in such feature subset. For each feature subset $F_{i}$, an SVM classifier can be built on all samples, which are represented by features from this feature subset. 10-fold cross-validation is adopted to evaluate the performance of such classifier. After all SVM classifiers have been evaluated, the classifier with the best cross-validation classification performance is picked up and the corresponding feature subset is selected as the optimum feature set. Features in such set are termed as optimum features (i.e., optimum methylation sites or genes).

### 4.3. SVM 

SVM is a statistics-based supervised model that identifies a hyperplane with a maximum margin between two groups of samples. When a number of samples from two classes are given, a separation line can be used to separate them. However, in most cases, the samples are not linearly separable in a low-dimensional space. Considering that samples will be easily separated in a high-dimensional space, the data are first mapped into a high-dimensional space via kernel trick to make them linearly separable. SVM is a powerful approach to implement such concept and model. 

In this study, classifying samples from four breast cancer subtypes is required, which is a multi-class classification problem. Thus, a multi-class SVM was trained by using a one-versus-rest strategy, in which multiple binary SVMs are constructed. Each binary SVM is separately trained on the positive samples from one class (e.g., one breast cancer subtype) and the negative samples from other classes (e.g., remaining breast cancer subtypes). For a query sample, each binary SVM gives the probability of it belonging to the corresponding class. The class with the highest probability is assigned to the sample. To quickly implement the SVM, a tool “SMO” in Weka [112] is employed in this study. John Platt’s sequential minimal optimization algorithm [113,114] is used to optimize the training procedures. We selected the polynomial function as the kernel. 

### 4.4. Synthetic Minority Over-Sampling Technique

As indicated in Section 4.1, sample sizes in different breast cancer subtypes are of great difference. The biggest subtype has more than 3.5 times the samples of the smallest subtype. In this case, a perfect classifier is difficult to build because the predicted results are apt to the biggest subtype. To tackle such problem, Synthetic Minority Over-sampling Technique (SMOTE) [115] is employed in this study. In such method, new samples are constructed and added into all classes except the largest class. In detail, let *x* be a sample in a minority class. Its Euclid distances to all other samples in this class are computed. Accordingly, *k* nearest neighbors can be found, where *k* is a pre-defined parameter. Randomly select one neighbor, say *y*, and construct a new sample *z*, which is defined as the linear combination of *x* and *y*. Because *x* and *y* are all in the same class and the new sample *z* has strong associations with them, *z* is more likely to be in such class and is added into this class. 

In this study, we use the tool “SMOTE” in Weka [112], which implements the above-mentioned SMOTE method. For subtypes: basal, Her and LumB, several new samples are constructed via “SMOTE” and added to each subtype. Finally, each subtype contained 120 samples. 

### 4.5. Performance Measurement

In this study, we used multi-class classifier to discriminate samples from four breast cancer subtypes. We evaluated the trained multi-class classifiers by using a 10-fold cross-validation [39,40,116,117,118]. For the predicted results yielded by 10-fold cross-validation, the sensitivity and specificity for each class were calculated. In addition, the overall accuracy and Matthews correlation coefficient (MCC) [119,120,121] were also computed to measure the classification performance. When defining *X* as the binary matrix of the predicted labels and *Y* as the binary matrix of the true labels, the MCC is calculated as follows:

(4) $$MCC=\frac{\mathrm{cov}(X,Y)}{\sqrt{\mathrm{cov}(X,X)\mathrm{cov}(Y,Y)}}=\frac{\sum_{i=1}^{n}\sum_{j=1}^{C}\left(x_{ij}-{\bar{x}}_{j})(y_{ij}-{\bar{y}}_{j}\right)}{\sqrt{\sum_{i=1}^{n}\sum_{j=1}^{C}{\left(x_{ij}-{\bar{x}}_{j}\right)}^{2}\sum_{i=1}^{n}\sum_{j=1}^{C}{\left(y_{ij}-{\bar{y}}_{j}\right)}^{2}}}$$

where cov(*X*,*Y*) represents the correlation coefficent of *X* and *Y*, ${\bar{x}}_{j}$ and ${\bar{y}}_{j}$ are the mean values in the *j*-th columns of *X* and *Y*, respectively, *n* represent the total number of samples and *C* indicates the number of labels. 

### 4.6. Enrichment Analysis

We mapped the selected methylation probes onto genes based on the probe annotation file of Illumina HumanMethylation450 BeadChip downloaded from https://www.ncbi.nlm.nih.gov/geo/query/acc.cgi?acc=GPL13534. These mapped genes were enriched onto GO and KEGG by using the Hypergeometric test. *p*-value was adjusted as false discovery rate (FDR). The GO terms and KEGG pathways with FDR smaller than 0.05 were considered significantly enriched biological functions. 

## 5. Conclusions

This study investigated the methylation profiles of patients of four breast cancer subtypes with several machine learning algorithms. Some functional genes with different methylation status in different subtypes were identified. These genes can be latent biomarkers or can be used to build an efficient predictor for the identification of different breast cancer subtypes.

## Figures and Tables

**Table 1:** Performance and optimum number of features of incremental feature selection (IFS) with support vector machine (SVM) and random forest (RF).

Terms	Sensitivity/Specificity	SVM	RF
Number of optimum features	/	1890	840
Matthews correlation coefficient (MCC)	/	0.925	0.860
Overall accuracy	/	0.949	0.906
Basal	Sensitivity	1.000	1.000
	Specificity	1.000	0.991
Her2	Sensitivity	0.973	0.892
	Specificity	0.986	0.982
LumA	Sensitivity	0.942	0.917
	Specificity	0.963	0.918
LumB	Sensitivity	0.921	0.841
	Specificity	0.974	0.963

**Table 2:** Performance and optimum number of features of IFS with SVM and RF on the dataset without LumB samples.

Terms	Sensitivity/Specificity	SVM	RF
Number of optimum features	/	480	40
MCC	/	0.961	0.951
Overall accuracy	/	0.979	0.974
Basal	Sensitivity	1.000	0.971
	Specificity	1.000	0.994
Her2	Sensitivity	0.973	0.946
	Specificity	0.981	0.987
LumA	Sensitivity	0.975	0.983
	Specificity	0.986	0.972

## Supplementary Materials

The following are available online at https://www.mdpi.com/1422-0067/20/17/4269/s1.
