# Supplementary material for: Identifying Methylation Pattern and Genes Associated with Breast Cancer Subtypes
Source: Int J Mol Sci. 2019 Aug 31;20(17):4269. doi: 10.3390/ijms20174269 (PMC6747348; doi:10.3390/ijms20174269)
Supplement: Supplementary file 1 [file ijms-20-04269-s001.zip › Supp_S9.docx]

**Supplementary Material S9:** The confusion matrices yielded by the SVM with top 10-100 features on the whole dataset.

1. **Confusion matrix for top 10 features**

|  |  | **Predicted label** | | | |
| --- | --- | --- | --- | --- | --- |
| **True label** |  | **Basal** | **Her2** | **LumA** | **LumB** |
|  | **Basal** | 31 | 1 | 2 | 0 |
|  | **Her2** | 1 | 20 | 6 | 10 |
|  | **LumA** | 0 | 31 | 24 | 65 |
|  | **LumB** | 0 | 11 | 11 | 41 |

1. **Confusion matrix for top 20 features**

|  |  | **Predicted label** | | | |
| --- | --- | --- | --- | --- | --- |
| **True label** |  | **Basal** | **Her2** | **LumA** | **LumB** |
|  | **Basal** | 31 | 1 | 2 | 0 |
|  | **Her2** | 1 | 29 | 6 | 1 |
|  | **LumA** | 1 | 5 | 72 | 42 |
|  | **LumB** | 0 | 7 | 12 | 44 |

1. **Confusion matrix for top 30 features**

|  |  | **Predicted label** | | | |
| --- | --- | --- | --- | --- | --- |
| **True label** |  | **Basal** | **Her2** | **LumA** | **LumB** |
|  | **Basal** | 32 | 1 | 1 | 0 |
|  | **Her2** | 1 | 32 | 3 | 1 |
|  | **LumA** | 1 | 5 | 88 | 26 |
|  | **LumB** | 0 | 2 | 15 | 46 |

1. **Confusion matrix for top 40 features**

|  |  | **Predicted label** | | | |
| --- | --- | --- | --- | --- | --- |
| **True label** |  | **Basal** | **Her2** | **LumA** | **LumB** |
|  | **Basal** | 32 | 1 | 1 | 0 |
|  | **Her2** | 1 | 32 | 1 | 3 |
|  | **LumA** | 1 | 5 | 85 | 29 |
|  | **LumB** | 0 | 1 | 18 | 44 |

1. **Confusion matrix for top 50 features**

|  |  | **Predicted label** | | | |
| --- | --- | --- | --- | --- | --- |
| **True label** |  | **Basal** | **Her2** | **LumA** | **LumB** |
|  | **Basal** | 32 | 1 | 1 | 0 |
|  | **Her2** | 2 | 33 | 1 | 1 |
|  | **LumA** | 1 | 4 | 95 | 20 |
|  | **LumB** | 0 | 3 | 16 | 44 |

1. **Confusion matrix for top 60 features**

|  |  | **Predicted label** | | | |
| --- | --- | --- | --- | --- | --- |
| **True label** |  | **Basal** | **Her2** | **LumA** | **LumB** |
|  | **Basal** | 33 | 0 | 1 | 0 |
|  | **Her2** | 2 | 33 | 1 | 1 |
|  | **LumA** | 1 | 4 | 99 | 16 |
|  | **LumB** | 0 | 4 | 13 | 46 |

1. **Confusion matrix for top 70 features**

|  |  | **Predicted label** | | | |
| --- | --- | --- | --- | --- | --- |
| **True label** |  | **Basal** | **Her2** | **LumA** | **LumB** |
|  | **Basal** | 34 | 0 | 0 | 0 |
|  | **Her2** | 1 | 35 | 1 | 0 |
|  | **LumA** | 2 | 2 | 99 | 17 |
|  | **LumB** | 0 | 1 | 12 | 50 |

1. **Confusion matrix for top 80 features**

|  |  | **Predicted label** | | | |
| --- | --- | --- | --- | --- | --- |
| **True label** |  | **Basal** | **Her2** | **LumA** | **LumB** |
|  | **Basal** | 34 | 0 | 0 | 0 |
|  | **Her2** | 1 | 35 | 1 | 0 |
|  | **LumA** | 1 | 5 | 95 | 19 |
|  | **LumB** | 0 | 1 | 14 | 48 |

1. **Confusion matrix for top 90 features**

|  |  | **Predicted label** | | | |
| --- | --- | --- | --- | --- | --- |
| **True label** |  | **Basal** | **Her2** | **LumA** | **LumB** |
|  | **Basal** | 34 | 0 | 0 | 0 |
|  | **Her2** | 1 | 35 | 1 | 0 |
|  | **LumA** | 0 | 4 | 97 | 19 |
|  | **LumB** | 0 | 1 | 15 | 47 |

1. **Confusion matrix for top 100 features**

|  |  | **Predicted label** | | | |
| --- | --- | --- | --- | --- | --- |
| **True label** |  | **Basal** | **Her2** | **LumA** | **LumB** |
|  | **Basal** | 34 | 0 | 0 | 0 |
|  | **Her2** | 1 | 35 | 1 | 0 |
|  | **LumA** | 0 | 5 | 101 | 14 |
|  | **LumB** | 0 | 0 | 19 | 44 |
